# Unilateral unusual branching pattern of the axillary artery: a case report

**DOI:** 10.1590/1677-5449.20250188

**Published:** 2026-08-28

**Authors:** Mamta Bishnoi, Monica Baxla, Swati Yadav, Vidhu Dhawan

**Affiliations:** 1 Santosh Medical College and Hospital, Ghaziabad, Uttar Pradesh, India.; 2 ESI Medical College, Alwar, Rajasthan, India.

**Keywords:** axillary artery, axillary region, anatomical variation, artéria axilar, região axilar, variação anatômica

## Abstract

The axillary artery is the continuation of the subclavian artery in the upper limb on both the sides of the human body. The axillary artery gives off six branches along with two terminal branches, but manifests various branching patterns, knowledge of which is useful for clinicians. A formalin fixed donated cadaver was used and the axillary region was dissected according to Cunningham’s Manual of Practical Anatomy. This case report describes a unilateral common trunk arising from the second part of the axillary artery, giving off lateral thoracic, posterior circumflex humeral, circumflex scapular, and thoracodorsal arteries. The present study also presents an embryological interpretation of the current variation. These variations are accidental findings discovered during dissection, but knowledge of them is useful for clinicians performing any surgical procedures in the axillary region.

## Resumo

A artéria axilar é a continuação da artéria subclávia no membro superior, em ambos os lados do corpo humano. A artéria axilar dá origem a seis ramos, além de dois ramos terminais, mas apresenta padrões de ramificação variados, cujo conhecimento é útil para os clínicos. Utilizou-se um cadáver doado, fixado em formalina, e a região axilar foi dissecada de acordo com o Cunningham’s Manual of Practical Anatomy. Este relato de caso descreve um tronco comum unilateral originado da segunda parte da artéria axilar, que, por sua vez, dá origem às artérias torácica lateral, circunflexa umeral posterior, circunflexa da escápula e toracodorsal. O presente estudo também apresenta uma interpretação embriológica da variação observada. Essas variações são achados incidentais descobertos durante a dissecção, mas o conhecimento sobre elas é útil para médicos que realizam procedimentos cirúrgicos na região axilar.

## INTRODUCTION

In humans, the subclavian artery originates from the arch of aorta on the left and from the brachiocephalic trunk on the right. It continues as the axillary artery (AA), after crossing the outer border of first rib as it enters the axilla, until the inferior border of the teres major muscle. Beyond the teres major, the AA continues as the brachial artery. The axillary artery is a major blood vessel supplying the lateral part of the thorax, the axillary region, and the whole of the upper limb.

Anatomically, the axillary artery is divided into three segments by the pectoralis minor muscle, and each segment gives off branches which supply the pectoral region, lateral part of the thoracic wall, the shoulder girdle, and the breast. The AA gives off six branches. The superior thoracic artery arises from the first segment, the lateral thoracic and thoraco-acromial arteries arise from the second, and the subscapular artery (SSA) and the anterior and posterior circumflex humeral arteries arise from the third segment of the axillary artery.^1^ The subscapular artery is the largest branch of the AA and gives off two terminal branches: the circumflex scapular artery (CSA) and the thoracodorsal artery (TDA).

Despite extensive documentation of arterial variants, routine dissections still reveal variant arterial patterns, meeting a need for knowledge of such variations for correct treatment and diagnosis by surgeons and radiologists managing axillary artery occlusion and quadrilateral space syndrome.^2^ In the present case, we report another such variation, because knowledge of variant forms of branching pattern of the axillary artery is necessary for clinicians to avoid iatrogenic complications.

The present study was carried out on cadavers donated to the department with written and informed consent for carrying out whole body dissection for educational and research purposes. All norms related to use of human cadavers in teaching and research were followed strictly as per the institutional guidelines. We also declare that the manuscript was prepared in accordance with the Helsinki Declaration.

## CASE DESCRIPTION

**Figure 1:**
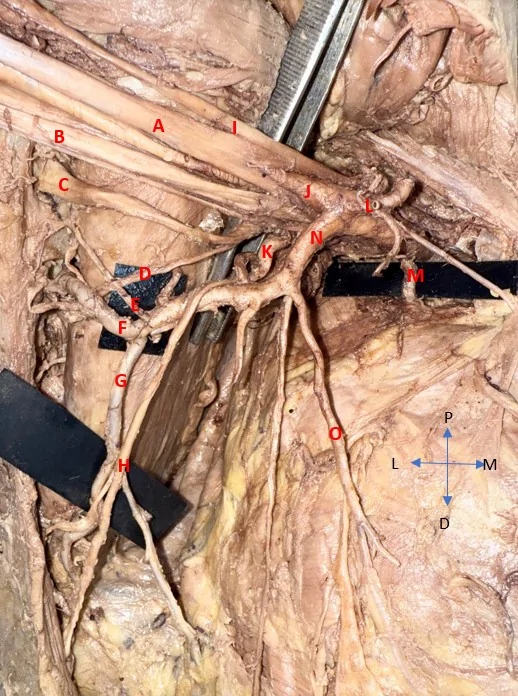
Showing the variation in the branching pattern of the axillary artery. A = median nerve; B = ulnar nerve; C = axillary nerve; D = upper subscapular nerve; E = posterior circumflex humeral artery; F = circumflex scapular artery; G = thoracodorsal artery; H = thoracodorsal nerve; I = musculocutaneous nerve; J = axillary artery; K = thoraco-acromial artery; L = superior thoracic artery; M = branch from the axillary artery forming a common trunk; N = anterior circumflex artery; O = lateral thoracic artery.

**Figure 2:**
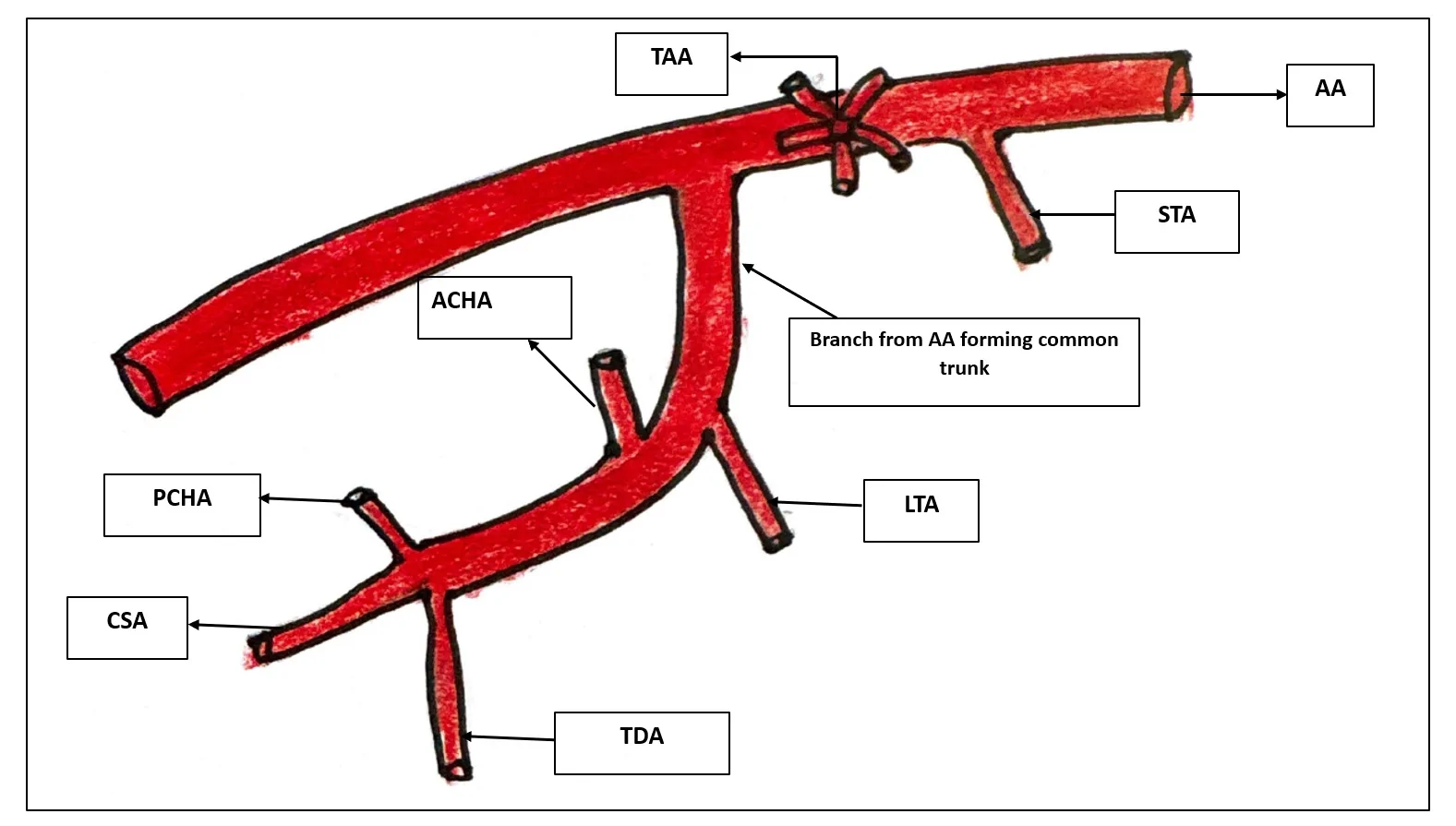
Schematic diagram showing the variation in the braching pattern of the axillary artery on the right side AA = axillary artery; ACHA = anterior circumflex humeral artery; CSA = circumflex scapular artery; LTA = lateral thoracic artery; PCHA = posterior circumflex humeral artery; STA = superior thoracic artery; TAA = thoraco-acromial artery; TDA = thoracodorsal artery.

During a routine dissection for undergraduates, the present variation was observed in a 60-year-old Indian male formalin fixed cadaver. Cunningham’s Manual of Practical Anatomy was followed for dissection of the axillary region. The branching patterns of the axillary arteries were different on each side. On the right side, once the structures had been cleared, the superior thoracic artery was seen arising from the first segment of the axillary artery. The thoraco-acromial artery arose from the second segment and gave off five branches. About 7 mm distal of the origin of the thoraco-acromial artery, a branch was seen arising from the axillary artery, whose circumference was almost the same as that of the main artery at the point of branching (2.8 cm and 2.6 cm for the AA and the branch from the AA, respectively). This branch gave off the lateral thoracic artery (LTA) from the medial side, which is usually a branch from the second part of the AA, and the anterior circumflex brachial artery from its lateral side. The artery travelled further, giving a muscular branch to the subscapularis and the posterior circumflex humeral artery (PCHA). The circumflex scapular and thoracodorsal arteries, which are the terminal branches of the subscapular artery, were observed to be arising from it as it reached the lateral border of the scapula, as shown in Figure 1 and illustrated schematically in Figure 2.

**Figure 3:**
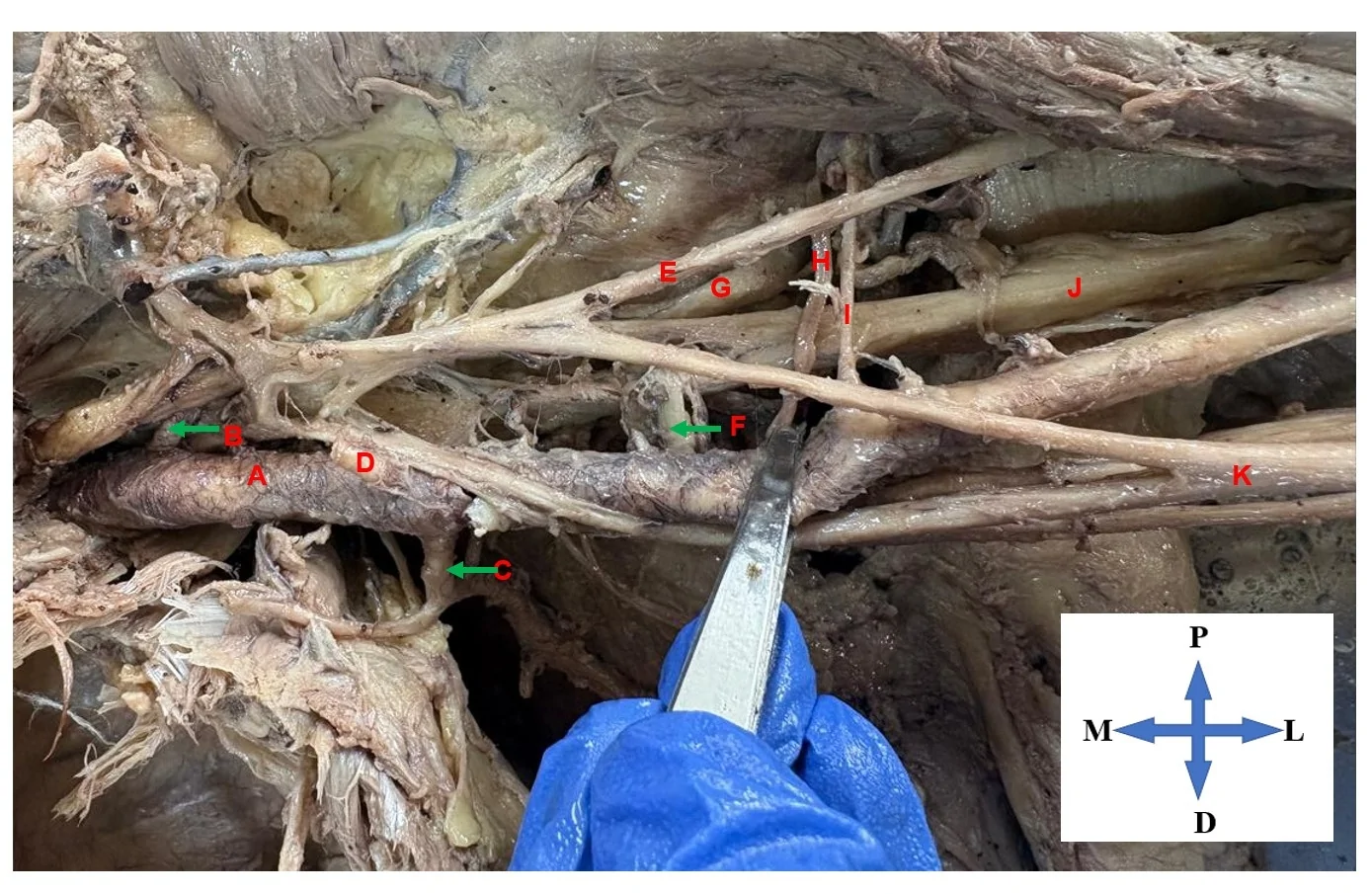
Shows normal branching pattern of the axillary artery. A = axillary artery; B = superior thoracic artery; C = lateral thoracic artery; D = thoraco-acromial artery; E = musculocutaneous nerve; F = subscapular artery; G = axillary nerve; H = posterior circumflex humeral artery; I = anterior circumflex humeral artery; J = radial nerve; K = median nerve.

**Figure 4:**
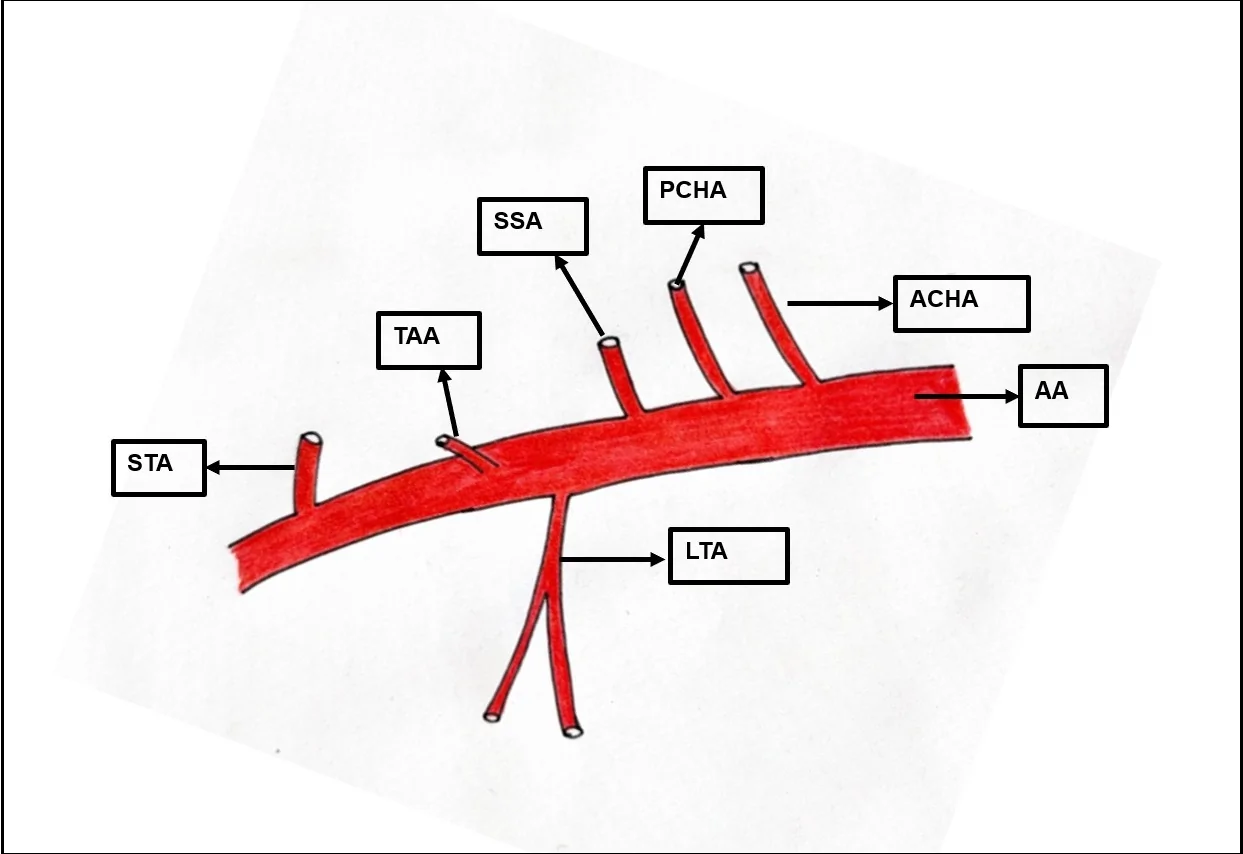
Schematic diagram showing normal branching pattern of the axillary artery on the left side. AA = axillary artery; ACHA = anterior circumflex humeral artery; SSA = subscapular artery; LTA = lateral thoracic artery; PCHA = posterior circumflex humeral artery; STA = superior thoracic artery; TAA = thoraco-acromial artery.

On the left side, the branches from the axillary artery were normal. The superior thoracic artery arose first, followed by the thoraco-acromial and lateral thoracic arteries. The subscapular artery was seen arising from the third part of the axillary artery and after a short distance the anterior and posterior circumflex humeral arteries were seen to be stemming out from the AA, as shown in Figure 3 with schematic illustration as shown in Figure 4. In the present study, the authors observed different AA branching patterns on the right and left sides

## DISCUSSION

The branching pattern of the axillary artery shows substantial variation. There are studies showing variability in origin as well as in branching pattern of the subscapular artery, which is the largest branch arising from the third part of axillary artery. Many authors have documented a common trunk of the subscapular artery along with any one or more branches of the axillary artery arising in common, as summarized in Table 1. The subscapular artery may arise from a common trunk along with the posterior circumflex humeral artery and sometimes the subscapular, circumflex humeral and profunda brachii arteries may arise from a common trunk.^20^

**Table 1:** Data summarizing previous authors’ findings.

**Authors**	**Year**	**Sample size**	**Findings**
Trotter et al.^3^	1930	384	36 (9.37%) – CT for LTA and SSA
			51 (13.28%) – CT for PCHA and SSA
Huelke^4^	1959	178	45 (25.00%) - CT for LTA and SSA
			27 (15.20%) - CT for PCHA and SSA
Saralaya et al.^5^	2008	1	CT for CSA, TDA, PCHA, TAA, and LTA
Olinger and Benninger^6^	2010	166	7 (4.20%) - CT for LTA and SSA
			20 (12.00%) - CT for PCHA and SSA
Astik and Dave^7^	2012	80	16 (20.00%) - CT for LTA and SSA
			8 (10.00%) - CT for ACHA, PCHA, SSA, and PBA
Shantakumar and Rao^8^	2012	1	CT for SSA and LTA
Goldman et al.^9^	2012	1	CT giving rise to TAA, LTA, ACHA, PCHA, TDA, and CSA
Loukas et al.^10^	2014	840	32 (3.93%) - CT for LTA and SSA
Sarkar et al.^11^	2014	1	CT for SSA, TDA, PCHA, and LTA
Naidoo et al.^12^	2014	100	3 (3.00%) – CT for SSA, ACHA, and PCHA
			15 (15.00%) - CT for PCHA, LTA, and SSA
			2 (2.00%) - PCHA from SSA
Fontes et al.^13^	2015	24	13 (54.20%) - PCHA from SSA
Ojha et al.^14^	2015	60	8 (13.00%) – CT for SSA, ACHA, and PCHA
			8 (13.00%) - CT of PCHA, SSA, and LTA
Kanaka et al.^15^	2015	30	9(30%) CT for PCHA and SSA
			6(20%) CT for TAA and SSA
Dimovelis et al.^16^	2016	1	CT for SSA, TDA, CSA, LTA, and PCHA
Tverskoi et al.^17^	2018	1	CT for TDA, CSA, PF, ACHA, and PCHA.
Konarik et al.^18^	2020	423	97 (22.93%) - CT for PCHA and SSA
Hur et al.^19^	2024	1	CT for pectoral branch, LTA, and SSA (B/L)
**Present study**		1	CT – LTA, ACHA, PCHA, TDA, and CSA

CT = common trunk; LTA = lateral thoracic artery; SSA = subscapular artery; PCHA = posterior circumflex humeral artery; CSA = circumflex scapular artery ACHA = anterior circumflex humeral artery; TDA = thoracodorsal artery; TAA = thoraco-acromial artery; PBA = profunda brachii artery; B/L = bilaterally.

As can be observed (Table 1), many authors have documented various other branches of the AA sprouting from a common trunk to the subscapular artery, including, among others, the LTA, PCHA, ACHA, and/or thoracodorsal. In our case report, the superior thoracic and thoraco-acromial arteries arose as usual, but the LTA, PCHA, ACHA, TDA, and CSA all arose from a common trunk branching from the main AA. Since the thoracodorsal and circumflex scapular arteries, which are branches of the SSA, also arose from this common trunk, we can name this CT the subscapular common trunk, as suggested by previous authors.^4,15^

The axillary artery may be used in procedures like coronary artery bypass,^21^ as access for transcatheter aortic valve replacement,^22^ or as a donor vessel in reconstructive surgery,^23^ and is also important in breast surgery.^24^ Hence, documentation of different variations of the axillary artery is essential to advance research in this area.

### Embryological relevance

During intraembryonic life, blood vessels are formed by 2 processes: vasculogenesis and angiogenesis. Vasculogenesis refers to the formation of blood vessels by coalescence of angioblasts, whereas angiogenesis refers to formation of vessels due to sprouting from existing vessels.^25^ Another process, vascular intussusception (non-sprouting angiogenesis), also plays an important role, in which existing vessels split to give rise to new vessels.^26^ VEGF (vascular endothelial growth factor), PDEF (platelet derived growth factor), and TGF-β (transforming growth factor β) are responsible for promoting development of the vascular system.^25^ Angiopoietin -1 is a sprouting factor that attaches to its receptor Tie-2, present on endothelial cells (EC) where sprouting has to occur during angiogenesis.^27^ In the present case, it might be possible that Angiopoietin -1 was bound to Tie-2 on abnormal sites, causing sprouting of new vessels and leading to such variation in the branching pattern of the AA.

## CONCLUSION

A thorough understanding of arterial variations is essential to alleviate certain procedural risks, such as in fractures of the upper end of the humerus in the axillary region, to optimize patient outcomes.

## Data Availability

Data sharing does not apply to this article, as no data were generated or analyzed.
